# Has the COVID-19 pandemic affected out-of-hours presentations in CAMHS?

**DOI:** 10.1192/bjo.2021.845

**Published:** 2021-06-18

**Authors:** Sacha Evans

**Affiliations:** Mildred Creak Unit, Great Ormond Street Hospital

## Abstract

**Aims:**

The aim of this project was to look at whether the COVID-19 pandemic, specifically lockdown, has impacted out-of-hours presentations to Child and Adolescent Mental Health Services (CAMHS) in North Central and East London.

**Method:**

Specialist Registrars (SpRs) on the Royal London/Great Ormond Street CAMHS Higher Training Scheme are contacted for advice regarding all CAMHS presentations in the North Central and East London area. Responsibilities includes provision of advice to 6 hospitals (including 4 emergency departments) and 4 child and adolescent inpatient units. A record of all phone calls and call-outs, including Mental Health Act and Section 136 (S136) assessments are maintained and this study compares pre- and post-COVID-19 data to see if there are any differences in number of presentations, on-site assessments (including Mental Health Act and S136 assessments over 2019 and 2020.

**Result:**

Numbers of CAMHS presentations were lower in 2020 (mean 74 patients per month) compared with 2019 (60 patients per month). This was consistent across all months except October and December. The largest difference was seen in March: 109 patients presented in March 2019, compared with 55 in March 2020. This is also reflected in the number of assessments conducted on site. However, there do not appear to be differences in the numbers of Mental Health Act or S136 assessments undertaken over 2020, compared with 2019.

**Conclusion:**

CAMHS out-of-hours presentations dropped off significantly at the start of the COVID-19 pandemic in the UK, and in particular, with the first lockdown (March to July 2020). Specialist Registrars provided advice via telephone less frequently in 2020 compared with 2019, and were required to do fewer on-site assessments of children and young people presenting with mental health difficulties.

There were no significant differences in Mental Health Act or S136 assessments between the two years, however, these numbers are too small to make any meaningful conclusions.

It is likely that children and adolescents were less likely to present to emergency departments for assessment of their mental health difficulties during the COVID-19 pandemic, rather than this reflecting a true reduction in mental health difficulties.

Recommendations:

It is helpful to continue to monitor CAMHS out-of-hour presentations.

Trusts may want to consider alternative settings for providing emergency CAMHS assessments, for example, mental health hubs.

Limitations:

This provision of data is subject to recall bias.

